# EEG-Based Brain-Computer Interface for Tetraplegics

**DOI:** 10.1155/2007/23864

**Published:** 2007-09-19

**Authors:** Laura Kauhanen, Pasi Jylänki, Janne Lehtonen, Pekka Rantanen, Hannu Alaranta, Mikko Sams

**Affiliations:** ^1^Laboratory of Computational Engineering, Helsinki University of Technology, 00280 Helsinki, Finland; ^2^Robotics Research Group, Department of Engineering Science, Oxford University, Oxford OX1 3PJ, UK; ^3^ORTON Orthopaedic Hospital, Invalid Foundation, 00280 Helsinki, Finland; ^4^Käpylä Rehabilitation Centre, Finnish Association of People with Mobility Disabilities, 00251 Helsinki, Finland

## Abstract

Movement-disabled persons typically require a long practice time to learn how to use a brain-computer interface (BCI). Our aim was to develop a BCI which tetraplegic subjects could control only in 30 minutes. Six such subjects (level of injury C4-C5) operated a 6-channel EEG BCI. The task was to move a circle from the centre of the computer screen to its right or left side by attempting visually triggered right- or left-hand movements. During the training periods, the classifier was adapted to the user's EEG activity after each movement attempt in a supervised manner. Feedback of the performance was given immediately after starting the BCI use. Within the time limit, three subjects learned to control the BCI. We believe that fast initial learning is an important factor that increases motivation and willingness to use BCIs. We have previously tested a similar single-trial classification approach in healthy subjects. Our new results show that methods developed and tested with healthy subjects do not necessarily work as well as with motor-disabled patients. Therefore, it is important to use motor-disabled persons as subjects in BCI development.

## 1. INTRODUCTION

A brain-computer interface (BCI) enables the control of applications based on brain signals, measured invasively or noninvasively. BCIs can help severely motor-disabled persons to communicate and control their environment. Single-EEG epochs measured during a few different mental or real tasks can be classified accurately enough to be translated into simple computer commands (for reviews see [1, 2]). Unfortunately, for successful performance, subjects often need several weeks or even months of training [3–5]. If learning takes place very slowly, it can decrease the motivation and willingness to use BCIs.

When advanced machine learning techniques are used, a BCI can learn to recognize signals generated by a novice user after less than one hour training period (see, e.g., [6–10]). Many of these analysis techniques have only been tested with offline data, or data are first collected in a 5–20 minutes calibration session without feedback and the classifier is then trained with this data and used in the next session where feedback is presented. Therefore, subjects receive either no feedback, or feedback is not up to date. Vidaurre et al. [11] used an alternative approach, in which they trained the classifier online with correct class labels during the feedback sessions. Their model was, however, never tested without supervised learning. Therefore, its performance in a BCI application could not be evaluated.

BCIs utilizing machine learning use various features of EEG signals as a basis for classification, such as P300 event-related potentials to visual or auditory stimuli [12], and EEG frequency patterns [13–16]. The most commonly used frequency pattern is the Rolandic mu-rhythm consisting of 10 Hz and 20 Hz frequency components recorded over the sensorimotor cortex [17]. These components are suppressed contralaterally during the movement execution [18, 19]. Another commonly used feature in healthy subjects is the slow premovement EEG potential called lateralized readiness potential (LRP/Bereitschaftspotential) [7, 20].

Paralyzed patients cannot move their extremities, but their sensorimotor cortices are activated during attempted movements. An fMRI study on five tetraplegic patients, paralyzed for 1–5 years due to spinal cord injuries, showed that these patients' sensory and motor cortices are activated during attempted hand and foot movements [21]. Very similar activations were found in healthy control subjects during real movements. In another fMRI study of nine paraplegic patients having complete spinal cord injury between T6 and L1 (1 month–33 years), activation patterns during motor attempts resembled those of a control group performing the corresponding movements, but were weaker in the patients [22]. The activation patterns, however, differed more between motor imagery of the control group and the patients' motor attempts.

The aim of the present study was to develop and evaluate a two-command BCI that tetraplegic patients could learn to control after a short training period. Six patients participated in a 30-minute online BCI experiment. The task was to move a circle on a computer screen by attempting either right- or left-hand movements every two seconds after a cue stimulus. Feature extraction and classification methods were first tested on healthy subjects performing real finger movements [23]. The classifier was trained after each movement attempt using the correct class labels. This enabled online feedback to the subjects already after the first ten trials (*∼*20 seconds from the beginning of the experiment). In applications, subject's intent cannot be directly known and thus supervised learning is impossible. Therefore, the classifier was not trained when testing the BCI performance.

## 2. MATERIAL AND METHODS

### 2.1. Subjects

Six male tetraplegic subjects participated in the study (Table 1). Neurological level C5 corresponds to the elbow flexors, level C6 to the wrist extensors, and level C7 to the elbow extensors. Subject S4 reported being left handed and the rest right handed. The tetraplegia in S3 was caused by Guillain-Barre syndrome, and in the rest of the subjects by trauma-induced spinal-cord injury (SCI). All the subjects, interviewed one week before they participated in the study, were volunteers and were not paid for their participation. They were all highly motivated and interested in the experiment.

The study was approved by the ethical Committee of the Hospital district of Helsinki and Uusimaa. The subjects were assisted to sign their informed consent to participate in the study.

### 2.2. Experimental setup

The experiment was performed with the BCI system developed at the Helsinki University of Technology (TKK-BCI) [24].

**Figure 1:**
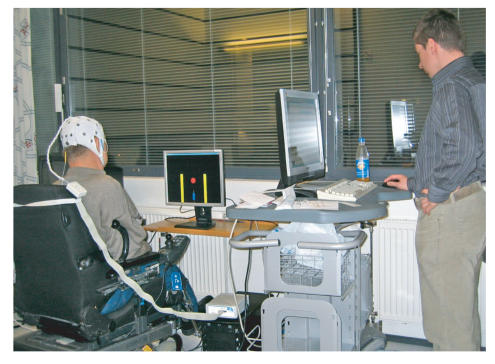
A tetraplegic subject using the BCI in his patient room at the rehabilitation centre. The BCI controller is on the subject's right side. The subject's task was to move the red circle to the yellow target on the side of the screen.

Subject's task was to move a circle from the centre of the computer screen to the target located on the left or right side of the screen by means of EEG signals related to attempted right- or left-hand movements (Figure 1). The subjects were instructed to attempt fast movements. They were shown movement examples which included finger lifting and pinching, and fist closing. The subjects were instructed to chose one of the movements and use it during the whole experiment. Subjects S1, S2, S4 attempted to close their fists, subject S5 attempted to lift his index fingers, and subjects S3 and S6 attempted to pinch their index finger and thumb together. The subjects were unable to move the body parts they attempted to move.

The experiment consisted of 6–20 seconds long games. A game started with an arrow indicating which target the subjects should try to reach with the circle, that is, which hand they were to attempt to move during the game. After the disappearance of the arrow, the circle appeared in the middle of the screen and two targets on both of its sides (Figure 1). A visual trigger was displayed below the circle. This trigger was a rectangle that decreased in size until it disappeared 0.8 second after its appearance. The subjects were instructed to attempt the movement when this trigger disappeared; this timing is later referred to as the cue. The gradually diminishing trigger enabled the subjects to prepare for the movements. Each attempted movement is called a trial. The rectangle re-appeared every 2 seconds (trial ISI = 2 seconds). A game consisted of 3–10 trials and lasted 6–20 seconds; there were short 2-second breaks between the games. If the trial was classified correctly, the circle moved to the direction of the correct target, otherwise it moved to the opposite direction. The game ended when the subject reached one of the targets, or a maximum of 10 trials was exceeded. It was also possible to reach the wrong target if enough trials were classified incorrectly. The subjects were instructed to fixate on the middle of the trigger during the games. Thus, the visual view was identical between the left and right tasks.

Based on a suggestion of subject S1, the game was modified for S2–S6. In the new version, the circle moved proportionally to the class probability given by the classifier: (P−.5)⋅k, where P is the output of the classifier, that is, the posterior probability of the most probable class given the model and the training data, and *k* is a distance measure in pixels adjusted according to the size of the screen. In other words, the higher the probability predicted by the classifier the longer the step the circle moved.

**Figure 2:**
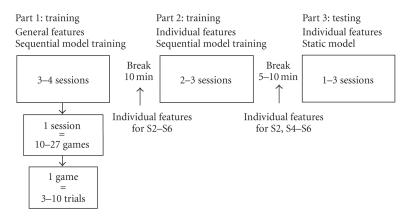
The structure of the experiment. The experiment consisted of three parts. Each part consisted of 1–4 sessions, each session of 10–27 games, and each game of 3–10 trials.

Figure 2 displays the overall structure of the experiment. Data was collected in 3.5–4 minutes sessions. There were approximately one-minute breaks between the sessions to avoid subjects getting tired. Each session consisted of 10–27 games, depending on how quickly the subjects hit the targets. The whole experiment contained three parts each consisting of one to four sessions depending on how the subjects felt and how well they performed. Longer breaks were kept between the three parts, during which individual EEG features were defined for each subject.

### 2.3. Recording

The experiments were conducted in patient rooms at the Käpylä Rehabilitation Centre in Helsinki (Figure 1). The patient was sitting in a wheelchair in front of the computer screen. During the measurements, one to three additional people were in the room. To decrease electrical interferences, lights, TV, and electrical beds were turned off. The data acquisition and BCI software were run on a 3 GHz, Pentium 4 PC.

Recordings were made with a 32-channel EEG electrode cap and amplifier. EEG was measured from 14 locations of the international 10–20 system: Fp1, Fp2, F3, F4, C3, C4, Cz, Fc1, Fc2, Cp1, Cp2, Fc5, Fc6, Fz. Horizontal and vertical eye movements were measured with three additional electrodes. Two of them were located at the outer canthi of the left and the right eye and the third one below the left eye. All electrodes were referenced to an electrode located in the middle of electrodes Cz and Fz. Electrode impedances, checked in the beginning of the experiment and during the longer breaks, were below 10 kOhm. The sampling frequency was 500 Hz and passband 0.1–225 Hz.

### 2.4. Features and classification

**Figure 3:**
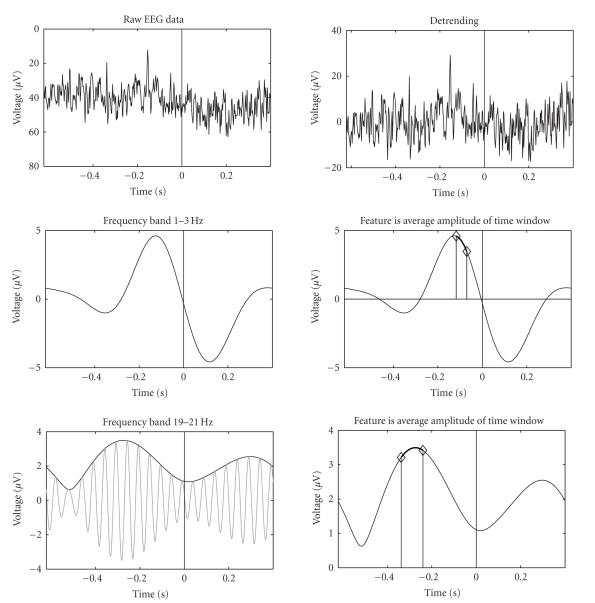
Computation of the features. The raw EEG signals were first preprocessed and detrended. The frequency components were extracted using fast Fourier transform. For frequencies over 3 Hz, the instant amplitude of the signal is taken using Hilbert transform. The feature is the average amplitude of some time window (second and third rows, right).

The selection and computation of features as well as classification were done using the same methods as described in [24]; here we give a short overview of the methods. Figure 3 shows an example of the feature extraction process (S6, channel C4) for two different frequency bands. The disappearance of the visual trigger is marked with a vertical line. One-second long EEG trials (starting 0.6 seconds before and ending 0.4 seconds after the cue) were extracted from each channel. First, linear trends were removed from the raw signals. Fast Fourier transform (FFT) was computed for each channel [26]. Different frequency bands were filtered by adjusting the Fourier components outside the passband to zero. For the 1–3 Hz band, temporal features were extracted by computing the inverse FFT. For all bands above 3 Hz, for example, the 19–21 Hz band (bottom row), the instantaneous amplitude of the signal was computed with the Hilbert transform [26]. The lower half of the two-sided spectrum was multiplied by two and the upper half was set to zero after which the magnitude of the inverse FFT was computed. The bottom left graph illustrates how the instantaneous amplitude follows the envelope of the fast varying filtered signal. The right second and bottom rows show how the actual features were computed from the signals by averaging amplitude values over short time windows.

In the first part of the experiment identical features were used for all subjects. Based on earlier studies with tetraplegics [27], the features were computed from the 1–3 Hz frequency band from seven adjacent 100 milliseconds time windows starting 400 milliseconds before and ending 300 milliseconds after the cue. Six electrodes over the sensorimotor cortex (C3, C4, Cp1, Cp2, Fc1, Fc2) were used. This gave a total of 42 features (six channels × seven time windows).

Due to good performance, the same set of features was used throughout the experiment with S1. During the first longer break, individual features were determined for S2–S6 from the data recorded in the first four sessions. For subjects S2 and S4–S6 new features were also determined during the second break based on the data from the previous three sessions.

For the feature selection process, a large set of features was computed from the training data using the same six channels. The features were computed from 2 Hz wide frequency bands starting from 1,2,…,38 Hz. Both 100 milliseconds and 50 milliseconds long, over lapping time windows starting from 400 milliseconds before and ending 300 milliseconds after the cue were investigated. Kolmogorov-Smirnov (KS) test statistic (see, e.g., [28]) was used as a difference measure between the class-conditional distributions for each feature independently. The KS test statistic is the maximum absolute difference between the cumulative distributions of two random variables (in this case the amplitude values related to the two tasks) describing how different the distributions are. All features were ranked according to the KS test statistic. When a particular frequency band and time window were chosen from one channel, the corresponding feature (the same band and time window) was also chosen from the rest of the channels. To decrease redundancies among the features, no overlapping frequency bands or time windows from the same channel were allowed. Seven different frequency-band and time-window combinations were included resulting in a total of 42 features.

Classification was based on several linear transformations of the feature space and a nonlinear logistic decision function. First, linear whitening transformation was used to reduce the dimensionality of the feature space and to produce uncorrelated features. This was applied to the whole data set, that is, data from both classes were combined. Second, three linear combinations of the whitened features to separate the classes were determined; Fisher's linear discriminant and the principal components corresponding to the largest variance for the both classes separately. Finally, a linear classifier with a logistic output function was used to transform the activation of the Fisher's discriminant and the magnitude of the feature vector in the directions of the principal components to class probabilities.

After feature extraction, each new feature vector was classified with the existing model. Based on the result, feedback was given to the subject. After each prediction, the oldest sample in the memory from the corresponding class was replaced with the new one and the classifier was updated with a maximum of 200 of these correctly labeled samples from both classes.

Online training of the model was started immediately after five samples (features) were acquired from each class. During the first ten trials of the experiment the circle was made to move in the correct direction. After that, the circle moved according to the prediction of the classifier and the user received visual feedback from his performance. Because supervised training of the classifier is not possible in real applications, the classifier was not updated in the testing part, in which the performance of the BCI was evaluated based on the classification accuracy.

## 3. RESULTS

### 3.1. Event-related potentials and features

**Figure 4:**
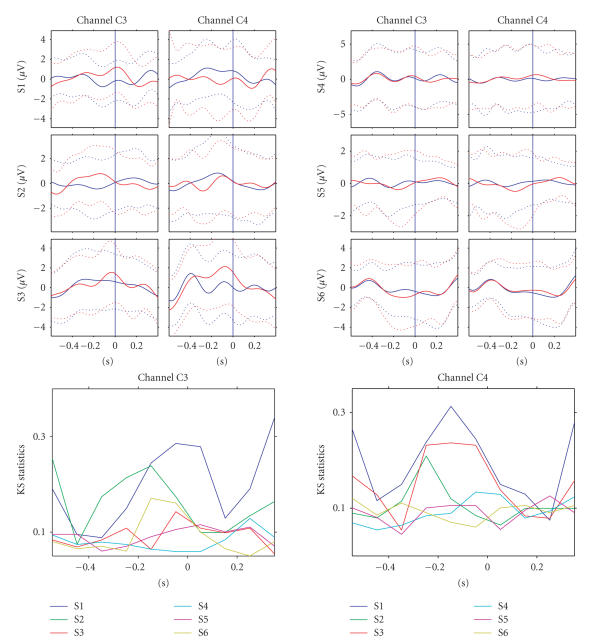
The upper part displays averaged signals ((± standard deviations, N*∼*150, filtered 1–3 Hz) for all subjects from electrodes C3 and C4 during both the right- (red) and left- (blue) attempted hand movement during the first part of the experiment. The blue vertical line indicates when the subjects were asked to perform the movement. The lower part of the figure shows the Kolmonogorov-Smirnov statistic between the classes of corresponding single trials.

**Figure 5:**
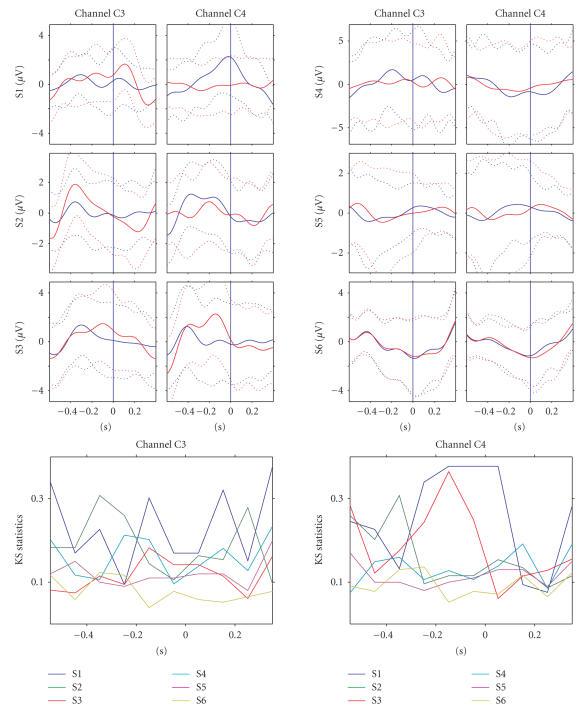
Displays averaged signals ((± standard deviations, N*∼*150, filtered 1–3 Hz) for all subjects from electrodes C3 and C4 during both the right- (red) and left- (blue) attempted hand movement during the first part of the experiment. The blue vertical line indicates when the subjects were asked to perform the movement.

The upper part of Figure 4 displays averaged signals (± standard deviations, passband 1–3 Hz) of all subjects in the training sessions at electrodes C3 and C4 during the attempted right- and left-hand movements (attempt onset indicated by the blue line). This activity was used as a feature in the first part of the experiment. The lower part of the figure shows how much the class-conditional distributions differ in the consecutive 100 milliseconds time windows according to the KS-statistic. Notice that the KS-statistic was calculated for the features, that is, amplitude values averaged over a 100 milliseconds time window (see Figure 3). The value of the test statistic is plotted for each feature in the middle of the corresponding time window. Channel C3 for S1, S2, and S6 and channel C4 for S1–S3 show a difference between the left- and right-attempted movements at several time points. Figure 5 shows the corresponding signals during the testing sessions. S1–S3 show rather similar patterns as during the training sessions but especially for S1 the class difference in C4 is more prominent.

The initial feature selection was not modified for S1 and thus seven adjacent time windows from the 1–3 Hz band were used as features throughout the experiment. Also, for S2 the first four and for S3 the first three selected features were from various precue time windows in the 1–3 Hz band. Each selected feature was taken from all eight channels leading to 24 and 18 features correspondingly. For S2, one feature was chosen from the 9–11 Hz band, the rest were close to the 20 Hz band. For S4–S6, the chosen features were widely distributed from 6 to 38 Hz; no features were chosen from the low frequency band.

### 3.2. BCI performance

BCI performance can be measured in two ways. We can measure how well subjects perform in the application. In the present experiment, this means how many times and how quickly the subjects were able to reach the target. We can also determine the classification accuracy of the single trials and the bit rate based on it.

Table 2 shows how well the subjects performed in the application. It shows the number of correct and incorrect hits in the last session in the test part of the experiment as well as the number of games in which the maximum number of trials was exceeded (maxes). Having perfect performance, the subjects could have hit the correct target in one session *∼*27 times. S1–S3 reached the correct target 8–15 times. S1 made no mistakes, and S2 and S3 each made one mistake. S4 had only 3 hits, but did not make any mistakes. Thus, these four subjects could make binary choices very accurately. However, the performance speed of S4 was slow because most games were not finished. The last column in the table displays how often, on average, subjects could hit the target per minute. This is calculated as a number of hits divided by the overall duration of the session. Thus, it includes time needed to display the arrow and time between the games. For S1–S4, these numbers are comparable to bit rates (bits/min) as they made very few mistakes. For example, S1 could make on average 3.8 binary choices per minute.

The two columns in the middle of Table 2 show the percentage of correct hits both with all games included (correct/games), and nonfinished games excluded (correct/hits), that is, including only games in which one of the targets was reached. The percentage of correct/games can be compared with classification accuracy, that is, how often the game was classified correctly, but note that it includes games that were not classified at all (maxes). The percentage of correct/hits reveals how many mistakes the subjects made. S5 had more misses than hits, and could not control the BCI. S4 had 100% accuracy, but he made only three hits, meaning that he performed accurately but very slowly.

### 3.3. Classification accuracy and bit rate of single trials

**Figure 6:**
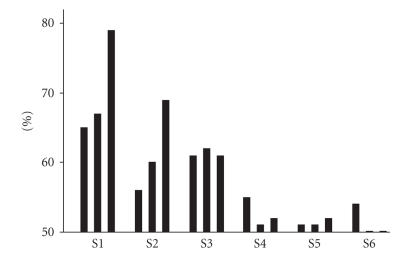
Part 1–3 average classification accuracy for subjects S1–S6.

The BCI performance was based on classification of EEG epochs related to single movement attempts. Assuming that these trials are independent, that is, EEG activation related to one movement attempt was unaffected by the previous movement attempts in the same game, we can calculate single trial classification accuracy and bit rate (Table 3). The number of single trials in the testing part, that is, that used to calculate the accuracy and bit rate is displayed in the column on the right; we rejected no trials due to artefacts. S1 achieved 79% mean accuracy. Although S2 and S3 were able to control the application with almost no mistakes, their mean classification accuracies were only 69% and 61%. Perelmouter and Birbaumer [29] suggest that a classification accuracy of *≈*70% is the requirement to control a binary language support program. S1 and S2 reached this criterion. The single trials of S4–S6 could not be classified above chance level. The bit rates, calculated as Wolpaw et al. [1], are shown per trial as well as per minute. The maximum bit rate per trial with two classes is 1 bit/trial. Predictions every two seconds result in a maximum of 30 bits per minute. The breaks between games were ignored because they depend on application. Subject S1 obtained a very high bit rate of 8 bits/min. The 3.1 per minute bit rate of S2 is still practical for control purposes, but one binary decision per minute of S3 is impractically slow. Subjects S1 and S2 had higher classification accuracies in the test part than in the beginning of the experiment (Figure 6). S3 and S4 did not improve their performance during the experiment.

To be able to exclude the possibility of BCI control based on eye movements, we simulated the experiment using only the EOG channels. Given the recorded data, this analysis is deterministic, that is, the single trial accuracies reported in the results section could be recovered by simulating the experiment with the given EEG channels. As in the online experiment, features were selected based on the data from the first training part, the classifier was optimized with the data from the second training part, and finally the single EOG trials of the test part were predicted with the obtained classifier. In the individual subjects, the offline single-trial classification accuracies were from 46% to 61% (mean 52%) for all subjects. S2 showed the highest classification accuracies of 61% in the last two testing sessions. These numbers are lower than the classification accuracies of the EEG channels.

## 4. DISCUSSION

Three out of six subjects (S1–S3) with complete tetraplegia learned to control a BCI after five to seven 4-minute training sessions. They moved a circle several times from the centre of the computer screen to the correct target on one of its sides. S1–S3 hit the target with an accuracy of 94%, 67%, and 57% (every game included), respectively. Despite the relatively low hit accuracy due to high number of games ending nonfinished, that is, ten trials were exceeded, these subjects made very few or no mistakes. The average correct hit rates were 2.2–3.8 hits/min. Assuming the single EEG trials independent, their attempted left- versus right-hand movements could be classified with mean accuracies of 79%, 69%, and 61% in a testing period when the classifier was no more trained. Transmitted information rate of the best subject (S1) was 8 bits/min.

S1 and S2 improved their performance during the experiment. Improvement was probably due both to the classifier adapting to subjects' signals and the subjects learning to control the BCI better. It is difficult to say to what extent the subjects learned control of their EEG signals in such a short training time. They might have learned to time their movement attempts better towards the end of the experiment. In addition, up-to-date feedback probably helped subjects to sustain concentration and try harder.

Real or attempted hand movements are appropriate for BCI use, because many tasks occur in body coordinates (moving a cursor or prosthesis). Only a few earlier EEG studies have examined how the sensorimotor cortex of tetraplegics reacts to movement imagery or attempts. Our recent magnetoencephalographic (MEG) and EEG studies showed that the sensorimotor rhythms of three tetraplegics (level of injury C4-C5; ASIA A classification) respond to attempted cued left- and right-hand finger movements [27]. In contrast to healthy subjects, the 10 and 20 Hz activity in these patients was not contralateral. Surprisingly, the best feature in the present experiment was the amplitude of the slow cortical brain activity (passband 0.5–3 Hz). It could be argued that these slow frequency features are related to eye movements or visual evoked potentials and not sensorimotor cortical activity. However, in the current experimental design we tried to ascertain that the subject's view was identical during both movement (left versus right) tasks; the cue was displayed in the center of the screen and the subjects were instructed to focus on it, not on the circle. The arrow indicating the correct target was presented before a game began and during the game, similar targets were displayed on both sides of the screen (Figure 1). To exclude the possibility that the trial classification was based on eye movements, we performed an offline analysis in which the trial classification was based on signals recorded by the EOG channels. Classification was on chance level in S1 and S3–S6. The classification accuracy of S2 was 61%, lower than 67% obtained on the basis of EEG channels. For S2, it is quite possible that the same features which were used in EEG trial classification were also picked up by the EOG channels. However, we cannot exclude the possibility that eye movements influenced the classification of his data.

Green et al. [30] measured 120 channel EEG while 24 tetraplegic patients attempted cued (ISI 7–10 seconds) finger and toe movements. Movement attempts evoked contralateral potentials in the motor cortex. We used the amplitude of the slow cortical brain activity (1–3 Hz) as initial features. These features were also chosen by the feature extraction algorithm for two good performing subjects (S2 and S3). We did not select features for S1 because he performed well with low-band signals as did subjects S2 and S3 who had several low frequency band features after the selection. In our previous studies, the best features for healthy subjects were nearly always in the 10 and 20 Hz range [24, 31] which was not the case for the present patients.

The methods for feature extraction, selection, and classification worked well in our previous study with ten healthy subjects [24]. Seven out of the ten healthy subjects could choose the correct target in 84–100% cases, 3.5–7.7 times a minute; their mean single trial classification rate was 79.6% and bit rate 10 bits/min. These results are much better than with the present tetraplegic subjects. The selected features also differed. In five healthy subjects the best features were around 10 Hz, in one around 20 Hz, and in four around 2 Hz. Especially, the contralaterality of the sensorimotor-cortex signals during attempted movements was not as clear as that with the healthy subjects performing real movements. The differences may be explained by two factors. First, the features were extracted around the visual trigger. Timing of single trials could jitter a lot and affect the low frequency features. Second, performing real movements is more natural than attempting movements. It is possible that tetraplegic subjects could improve their performance after more training; they could learn to produce more distinctive brain activations during the attempted movements and learn to time their movement attempts more accurately. As an example, Pfurtscheller et al. [32] showed that when one tetraplegic patient learned to control a hand orthosis by controlling his sensorimotor EEG by imagining a foot movement, the amplitude of mu-rhythm in his EEG increased over the five months of training.

In the present experiment, we used a supervised approach to classifier training during the training sessions [24]. Our approach has several advantages. First, a separate training session to determine the model parameters is unnecessary and feedback can be given almost from the beginning of the experiment. Second, subjects receive up-to-date feedback and can change their strategy in response to the feedback. Third, to give more informative feedback to subjects, the circle moved according to the posterior probability of the classifier for subjects S2–S6. The larger the class probability, the longer step the circle took. This informed the subjects about the EEG activity related to the current attempted movement compared to the previous ones that the model was trained with. It also made possible the low number of mistakes in the application, as consecutive wrong classifications with low probabilities did not result to miss. These features probably facilitated the learning of the BCI control.

It is difficult to compare our application performance with other studies because the game ended if the subject did not reach the target in ten trials. In addition, our single-trial bit rates are difficult to compare with those obtained by others, because we assume that the consecutive movement attempts are independent, which is not necessarily true. Most studies do not even show single trial accuracies. The use of BCIs by motor-disabled persons has been examined only in a few studies (see, e.g., [3, 33, 34]). Neuper et al. [35] trained a patient with infantile cerebral paresis over a period of 22 weeks, to use the “Graz-BCI”. The subject's task was to move an arrow or choose letters from either side of the screen during an eight-second long trial. The subject was trained to control the 20 Hz activity, using two mental tasks performed continuously for 4 seconds: imagination of right-hand movement and mental relaxation. After this 22-week extensive training period, the subject could choose one out of two letters with 70% accuracy. In another study, Pfurtscheller et al. [32] showed that one subject with a high-level spinal cord injury (C4/C5) could produce cue-stimulus dependent changes to the sensorimotor rhythms after five months of training; the patient could open a hand orthosis by imagining right hand movement.

The user group gaining the most from BCI technology are probably locked-in patients—not tetraplegics. The latter can use various switches such as head and eye mice, puff controls, and so forth. However, use of these methods can become tiresome after a long use. BCIs could offer an additional or alternative control method in producing text, and controlling environment and internet or email applications. In future, tetraplegics could use BCIs to control a hand prosthesis. Working with tetraplegics also provides us insight into how BCIs would work also with other motor-disabled groups.

In conclusion, we studied whether tetraplegic subjects could gain control of a BCI after a short training period. Our approach was based on recognition of features in single EEG trials without knowing the exact timing of the movement. Data from six electrodes is used. Model parameters could be trained quickly and no separate offline calibration session was needed. The results show that some tetraplegic subjects could learn to control a two-command BCI after only a short training period. Compared with a similar study performed with healthy subjects [24], our results show that methods developed and tested with healthy subjects do not necessarily work as well with motor-disabled patients. Therefore, it is important to use motor-disabled persons as subjects in BCI development.

## Figures and Tables

**Table 1:** The subjects' age, time since injury, and cite of injury, as well as the lowest site where some (albeit less than normal) movement could be detected are displayed.

Subject	Age	Time since injury	Cite of injury (movement detected)		ASIA [25]	Cause of tetraplegia
			Left	Right		
S1	46	4 yr.	C4(C4)	C4(C4)	A	SCI
S2	59	1.5 yr.	C4(C7)	C4(C7)	—	Syndroma Guillain Barre
S3	26	4 mo.	C4(C5)	C4(C5)	A	SCI
S4	47	4 mo.	C5(C4)	C5(C5)	B	SCI
S5	50	3 mo.	C5(C7)	C5(C7)	A	SCI
S6	63	35 yr.	C5(C6)	C5(C6)	B	SCI

**Table 2:** The number of games in which the subjects hit the correct/incorrect target (or exceeded maximum of ten trials) in the last session in the third part of the experiment (static model). The subjects did different amount of sessions depending on how tired they got. The percentage of games where the target was hit as well as the correct hits of all hits is displayed in the middle. The right-most column shows the correct hits/min calculated as the number of correct hits divided by the overall duration of the experiment.

Subject	Correct/incorrect (max) games	Correct games (%)	Correct hits (%)	Correct hits/min
S1	15/0 (1)	94	100	3.8
S2	10/1 (4)	67	91	2.7
S3	8/1 (5)	57	89	2.2
S4	3/0 (7)	30	100	0.9
S5	1/2 (8)	9	33	0.3
S6	9/5 (4)	50	64	2.4

**Table 3:** Theoretical classification accuracy, bitrate and bitrate/min in the third part of the experiment. The number of right/left single trials are displayed on the right.

Subject	R(%)	L(%)	Mean	Bitrate/trial	Bitrate/min	*#* trials right/left
S1	81	78	79	0.27	8.00	53/54
S2	71	66	69	0.1	3.12	104/104
S3	63	58	61	0.03	1.00	157/151
S4	55	49	52	0	0.04	94/105
S5	50	53	52	0	0.02	115/100
S6	45	53	50	0	0.00	154/163
